# Nanostructured Lipid Carriers (NLC)-Based Gel for the Topical Delivery of Aceclofenac: Preparation, Characterization, and *In Vivo* Evaluation

**DOI:** 10.3797/scipharm.1202-12

**Published:** 2012-06-18

**Authors:** Dilip Patel, Sandipan Dasgupta, Sanjay Dey, Y. Roja Ramani, Subhabrata Ray, Bhaskar Mazumder

**Affiliations:** 1 Department of Pharmaceutical Sciences, Dibrugarh University, Dibrugarh-786004, Assam, India.; 2 Department of Pharmacology, M. K. C. G. Medical College and Hospital, Berhampur, Orissa, India.; 3 Department of Pharmaceutics, Bengal School of Technology, Hoogly, West Bengal, India.

**Keywords:** Aceclofenac, Nanostructured lipid carriers (NLC), Topical gel, Nanoparticle

## Abstract

The aim of this study was to prepare nanostructured lipid carriers (NLC)-based topical gel of aceclofenac for the treatment of inflammation and allied conditions. Stearic acid as the solid lipid, oleic acid as the liquid lipid, pluronic F68 as the surfactant, and phospholipon 90G as the co-surfactant were used. NLCs were prepared by melt-emulsification, low-temperature solidification, and high-speed homogenization methods. Characterization of the NLC dispersion was carried out through particle size analysis, scanning electron microscopy (SEM), differential scanning calorimetry (DSC), and an *in vitro* release study. The anti-inflammatory effect of the NLC gel was assessed by the rat paw edema technique and compared to marketed aceclofenac gel. The NLC dispersions exhibited d_90%_ between 233 nm and 286 nm. All of the NLC showed high entrapment efficiency ranging from 67% to 82%. The particle size of NLC was further confirmed by the SEM study. The result of DSC showed that aceclofenac was dispersed in NLC in an amorphous state. Both the entrapment and release rate were affected by the percentage of oleic acid, but the method of preparation affected only the entrapment efficiency. The nanoparticulate dispersion was suitably gelled and assessed for *in vitro* permeation. Finally, NLC-based gels were found to possess superior (almost double) the anti-inflammatory activity compared to the marketed product. The anti-inflammatory activity of NLC gel showed a rapid onset of action, as well as a prolonged duration of action as compared with the marketed gel.

## Introduction

Nanostructure lipid carriers (NLC) are the new generation of lipid nanoparticles, attracting major attention as novel colloidal drug carriers for topical use. NLC were developed to overcome the limitations associated with the SLN. SLN consist of solid lipids, while NLC consist of a mixture of specially blended solid lipid (long chain) with liquid lipid (short chain), preferably in a ratio of 70:30 up to a ratio of 99.9:0.1. The resulting matrix of the lipid particle shows a melting point depression compared to the original solid lipid, however the matrix remains solid at body temperature [1]. Commonly observed disadvantages of SLN include limited drug-loading capacity, drug expulsion during storage, and relatively high water content in the dispersions (70–99.9%) [2, 3]. As compared to SLN, NLC have a higher drug-loading capacity for a number of active compounds, and avoid or minimize potential expulsion of active compounds during storage [2]. For a number of drugs, the solubility of liquid lipid is higher than that of solid lipid, which enhances drug-loading [4]. NLC possess numerous features that are advantageous for the topical route of application. These carriers are composed of physiological and biodegradable lipid, exhibiting low systemic toxicity and low cytotoxicity [5]. The small size of lipid particles ensure close contact to the stratum corneum and can enhance drug flux through the skin, and due to their solid lipid matrix, a controlled release from these carriers are possible [5, 6].

Aceclofenac is a potent analgesic, antipyretic, and anti-inflammatory agent. It has been approved for the treatment of various kinds of pain, osteoarthritis, and rheumatoid arthritis [7]. An arthritic condition demands a controlled-release drug delivery system for a prolonged period to satisfy the goals of the treatment like reducing pain and inflammation, slowing disease progression, and preventing adverse reactions. The designing of a topical drug delivery system of aceclofenac could not only increase delivery of drug locally and improve the release of the drug for a prolonged period, but also reduce the risk of gastrointestinal side effects and toxicity. The topical administration of aceclofenac could eliminate systemic side effects, which could also improve patient compliance [6, 8].

The present research explored the feasibility of NLC as a novel carrier system for the topical application of aceclofenac with regard to the modulation of release of aceclofenac. The *in vitro* permeation of the drug through rat skin and the anti-inflammatory activity of NLC gel were also evaluated.

## Materials and Methods

The following materials were used from the indicated sources, without further purification. Aceclofenac was procured as a gift sample from Cipla Ltd., Sikkim, India. Cetyl palmitate, stearic acid, tristearin, pluronic F68, carrageenan, dialysis membrane 70, carbopol 940P, xanthan gum, HPMC, and chitosan were purchased from Himedia, Mumbai, India. Phospholipon 90G (phosphotidylcholine 90%) was kindly gifted by GmbH, Germany. Oleic acid and isopropyl myristate were purchased from Loba Chemie, India. The other chemicals were of analytical reagent grade.

### Solid lipid selection

The selection of solid lipid was based on the solubility of aceclofenac to give a visibly clear solution in lipid, which melts under normal light when seen with the naked eye. The lipid**s** used for this study were stearic acid, cetyl palmitate, and tristearin. Aceclofenac (10 mg) and varying quantities of selected lipids were heated above the melting point of the lipid in a temperature-controlled water bath (Remi, Mumbai, India) in 15 ml glass vials. After melting the lipid in vials, the solubility of aceclofenac was observed under normal light [9, 10].

### Partitioning behavior of aceclofenac in various lipids

10 mg of aceclofenac was dispersed in a mixture of melted lipid (1 g) and hot distilled water (1ml). The mixture was shaken for 24hrs in a hot water bath at 65±5°C. After cooling, the aqueous phase was separated by ultracentrifugation (Remi, Mumbai, India) and the drug content was analyzed spectrophotometrically [11].

### Preparation of NLC

#### Melt-emulsification and low-temperature solidification method

The formulations of different ingredients are shown in Table 1. Aceclofenac and phospholipon 90G were dissolved in methanol and mixed with an acetone solution containing a blend of stearic acid and oleic acid. The mixtures were sonicated for 15 minutes and added dropwise to the pluronic F68 solution. After that, the mixture was stirred at 3000 rpm for 30 minutes at 70°C. The mixed solution was transferred to an icy water bath and stirred for 4 hours at 3000 rpm. The NLC dispersions were lyophilized for further study [12, 13].

#### Ultrasonication method or high-speed homogenizer method

The formulations of different ingredients are the same as the NLC, prepared by melt-emulsification and the low-temperature solidification method (Table 1). Aceclofenac and Phospholipon 90G were dissolved in methanol and mixed with an acetone solution containing a blend of stearic acid and oleic acid. The mixture was then added dropwise to pluronic F68 solution at 70°C. A pre-emulsion was obtained by homogenization using an Ultra-Turrax T25 (IKA-werke GmBH, Germany), at 15000 rpm for 10 minutes at 70°C. This pre-emulsion was ultrasonicated (20w) for 15 minutes to prevent the crystallization of lipids. The o/w emulsion obtained was subsequently cooled down to room temperature with continuous stirring, and the lipid was recrystallized to form NLC. The obtained NLC dispersions were lyophilized and used for further studies. [14, 15].

### Characterization of aceclofenac nanostructured lipid carriers

#### Particle size and particle size distribution

Particle size and polydispersity index (PI), which are the measures of the distribution of nanoparticle population, were determined by using Malvern Mastersizer 2000MU (Malvern, UK, detection limit 0.01–1,000 μm). The obtained data were evaluated using the volume distribution (d_10%_, d_50%_, d_90%_). The d_90%_ indicates that if the diameter 90% is registered as 1 μm, then 90% of particles have a diameter of 1 μm or less. The PI was measured by the span, which can be calculated from the following equation [16]. Increase in the span value indicates increase in PI.

Eq. 1 $$\text{SPAN}=\frac{\text{D}90\%-\text{D}10\%}{\text{D}50\%}$$

Where d_90%_ is the particle diameter at 90% of the cumulative size, d_10%_ is the particle diameter at 10% of the cumulative size, and d_50%_ is the particle diameter at 50% of the cumulative size.

#### Zeta potential (ζ)

The zeta potential of NLC was measured by using the Zetasizer 2000 (Malvern, UK). The NLC suspensions were diluted with double distilled water (1:100) to get a uniform dispersion prior to analysis. The conductivity of the diluted sample was measured to choose the detection model. The whole measurement was carried out at 25°C.

#### Scanning electron microscopy

The morphological characteristic of NLC was determined by a scanning electron microscope (JEOL-JSM-6360, Japan). One drop of sample was placed on a slide and excess water was left to dry at room temperature. The slide was attached to the specimen holder using double coated adhesive tape and gold coating under vacuum using a sputter coater (Model JFC-1100, JEOL, Japan) for 10 minutes, and then investigated at 20kV [17].

#### Drug entrapment efficiency

A volume of 2.0 ml of each drug-loaded sample was centrifuged (Microfuge, Remi motors, Mumbai) at 12500 rpm for 45 minutes to separate the lipid and aqueous phase. The supernatant was then diluted with methanol, filtered through 40μm filter paper (Hi-media, Mumbai) and the drug content was determined by the UV-VIS spectrophotometer (UV-1800, Shimadzu, Japan) at 273 nm. The entrapment efficacy of NLC was calculated as follows:

Eq. 2 $$\text{EE}=\left(\frac{\text{Wa}-\text{Ws}}{\text{Wa}}\right)\times 100$$

Eq. 3 $$\text{DL}=\left(\frac{\text{Wa}-\text{Ws}}{\text{Wa}-\text{Ws}+\text{Wl}}\right)\times 100$$

Where EE is entrapment efficiency, DL is Drug loading, Wa stands for the mass of aceclofenac added to the formulation, and Ws is the analyzed weight of the drug in supernatant and W*l* is the weight of lipid added [18, 19].

#### Differential scanning calorimetry (DSC)

DSC analysis of aceclofenac (ACLO), stearic acid (SA), pluronic F68 (PF68), physical mixture of stearic acid and oleic acid (SA+OA), and NLC formulation were performed using the Perkin-Elmer DSC instrument (Jade, USA). The instrument was calibrated with indium. Samples (5mg) were heated in aluminum pans under a dry nitrogen environment. The analysis was performed at a heating rate of 10°C/min between 20–240°C [20].

#### In vitro release study of NLC

The *in vitro* release studies were performed using Franz diffusion cells to evaluate the aceclofenac release profile from each formulation. Dialysis membrane 70 (Hi-Media, Mumbai, India) having pore size 2.4 nm and a molecular weight cut-off between 12,000–14,000, was used and mounted on the Franz diffusion cells. The surface area of the release membrane was 3.14 cm^2^. A phosphate buffer of saline (PBS) pH 7.4 was used as the receptor medium (45 ml) being stirred at 700 rpm. NLC dispersion (equivalent to 1mg of aceclofenac) was placed in the donor compartment. During the experiments, the solution in receptor side was maintained at 37±0.5°C. At predetermined time intervals, 3 mL of the samples were withdrawn from the receiver compartment and replaced by the same volume of freshly prepared PBS (pH 7.4). The samples were analyzed by the UV-Visible spectrophotometer (UV-1800, Shimadzu, Japan) at 273nm.

#### Preparation and evaluation of aceclofenac-loaded NLC gel

The suitable NLC formulation for the topical delivery of aceclofenac was selected based on the evaluation of characteristics like: particle size, entrapment efficiency, and *in vitro* release. It was found that the formulation F4 is more suitable among the other formulations. Different gelling agents such as: carbopol 940P, xanthan gum, chitosan, and hydroxypropyl methyl cellulose (HPMC) were used for the conversion of the NLC dispersion into the NLC gel formulation. Based on the compatibility with the NLC dispersion and the ease of spreadability, carbopol was selected as the gelling agent. The composition of different gel formulations are mentioned in Table 2. Carbopol was dispersed in the NLC dispersion using a mechanical stirrer (Remi, Mumbai, India) at a speed of 1200 rpm. The dispersion was neutralized using triethanolamine. The gel was allowed to stand overnight to remove entrapped air.

#### In vitro permeation study of NLC gel

The *in vitro* permeation studies of NLC gel were performed using the same method used for the *in vitro* release study of NLC dispersion, except for using rat skin instead of a dialysis membrane. The hairs on the stratum corneum of excised rat skin were removed and cleaned. The excised skin was placed facing the donor compartment, and the dermis faced the receptor compartment.

#### Analysis of permeation data

The permeation profiles were constructed by plotting the cumulative amount of aceclofenac permeated per unit area (μg/cm^2^) of skin versus time. The steady-state flux (Jss) of aceclofenac was calculated from the slope of the plot using linear regression analysis [21]. The permeability co-efficient (Kp) of the drug through the membrane was calculated using the following equation [22].

Eq. 4 $$\text{Kp}=\frac{\text{Jss}}{\text{C}}$$

Where C is the initial concentration of the drug in the donor compartment.

The penetration-enhancing effect was calculated in terms of the enhancement ratio (Er), using the following equation [23].

Eq. 5 $$\text{Er}=\frac{\text{Jss of formulation}}{\text{Jss of control}}$$

#### Skin irritation study

For the skin irritation study of the NLC gel formulation, rats were divided into four groups, each group containing three rats. Hairs were depleted from the back side of rats with the help of depilatories, and the area was marked on both sides. One side served as the control while the other side served as the test. Gel (500mg/rat) was applied once a day for seven days, and skin irritation from the formulation was determined by observations of any skin sensitivity and reactions such as redness, edema, and skin rash. The skin irritation effect of the gel was graded as: A-no reaction; B-slight, patchy erythema; C-moderate but patchy erythema; D-moderate erythema, and E-severe erythema with or without edema [24].

### In vivo anti-inflammatory study

The anti-inflammatory study was conducted on adult male Wister rats having a weight range between 130–150 g. The animals were divided into four groups, each group containing six animals. Group I received a topical saline application (Control group), group II received marketed Hifenac gel, group III received NLC F4 gel, and group IV received NLC F12 gel. The volume of paw edema (milliliter) was measured for each animal using a digital plethysmometer (Orchid Scientfics, Nashik, India). The rats were marked on the left hind paw just beyond the tibia-tarsal junction, and every time the paw was dipped in the electrolyte fluid column up to a fixed mark to ensure constant paw volume. The tested preparations were applied to the left hind paws of rats for 30 minutes before the induction of inflammation by carrageenan. The initial paw volume of the rats was measured just before carrageenan injection, and the increase in volume due to fluid displacement was noted from a digital display, followed by the injection of 0.1 ml of 1% (w/v) carrageenan solution in saline in the sub plantar region of left hind paws of the rats. Measurement of paw volumes was done after 1, 2, 4, 8, 12, and 24 h. The edema rate and inhibition rate of each group was calculated as follows:

Eq. 6 $$\text{Edema Rate }(\text{E}\%)=\frac{\text{Vt}-\text{Vo}}{\text{Vo}}\times 100$$

Eq. 7 $$\text{Inhibition Rate }(\text{I}\%)=\frac{\text{Ec}-\text{Et}}{\text{Ec}}\times 100$$

Where Vo is the mean paw volume before carrageenan injection (ml), Vt is the mean paw volume after carrageenan injection (ml), Ec is the edema rate of control group, and Et is the edema rate of the treated group.

Data were expressed as the mean ± S.D and statistically assessed by one-way analysis of variance (ANOVA). Values for the percentage edema rate for NLC-based gel and Hifenac gel (standard) were compared with the saline control and the differences were determined statistically using an appropriate post-hoc test (Dunnett’s t test). P<0.05 was considered as significant.

## Results and Discussion

### Selection of solid lipid

To develop a NLC system of poorly water-soluble aceclofenac, a selection of suitable solid lipid, liquid lipid, surfactant, and co-surfactant is very critical. All excipients under the GRAS (Generally Regarded as Safe) category were selected for the formulation of NLCs. The higher solubility of the drug in the solid lipid is important for the NLC formulation, to maintain the drug in solubilized form. To study the lipid solubility of aceclofenac, a range of solid lipids (stearic acid, cetyl palmitate, tristearin) were selected. Aceclofenac had the highest solubility in stearic acid compared to other lipids studied in this investigation.

### Partitioning behavior of aceclofenac in various lipids

Determination of the partitioning behavior of the drug is an important criterion, as it affects the entrapment efficiency as well as the release of the drug from the formulation. The partitioning behavior of aceclofenac in different lipids is presented in Table 3. The amount of drug in the aqueous phase was calculated using the calibration curve (Absorbance = 0.0240 × Concentration of aceclofenac, R^2^=0.9998) of aceclofenac in water. Aceclofenac showed higher partitioning in stearic acid than tristearin, followed by cetylpalmitate. In this study, stearic acid was selected as the solid lipid for the formulation of NLC because stearic acid has more potential to solubilize the aceclofenac as compared to the other two lipids.

### Preparation and characterization of NLC dispersion

The NLC dispersion was successfully prepared by melt-emulsification and the low-temperature solidification method, as well as by the high-speed homogenization method. The particle size distribution and span values of NLC are presented in Table 4. In particle sizing technology, span is a measure of the poly-dispersity index (PI), which represents the distribution of a nanoparticle population or the uniformity of distribution of nano-particles. Here it was observed that an increase in the amount of Phospholipon G and oleic acid led to a significant decrease in the span value of the formulations, because the particles separated in a stable form with no or only a slight degree of aggregation. A high span value indicates a wide distribution in sizes and a high polydispersity. The d_90%_ value for NLC was determined by using the Malvern Mastersizer (Hydro MU 2000, UK) and shows values ranging from 233 to 286 nm.

Zeta potential is essential for evaluating the storage stability of colloidal dispersions. The zeta potential of the different formulations was found within the range of −9.2 mV to −13.1 mV and −9.3 mV to −12.8 mV for the NLC prepared by melt-emulsification and low-temperature and high-speed homogenization methods, respectively. The zeta potential value of drug-loaded NLC was found to be lower than the blank NLC, and this might be due to the sharing of charge of the drug on the lipid matrix.

### Scanning electron microscopy

**Fig. 1:**
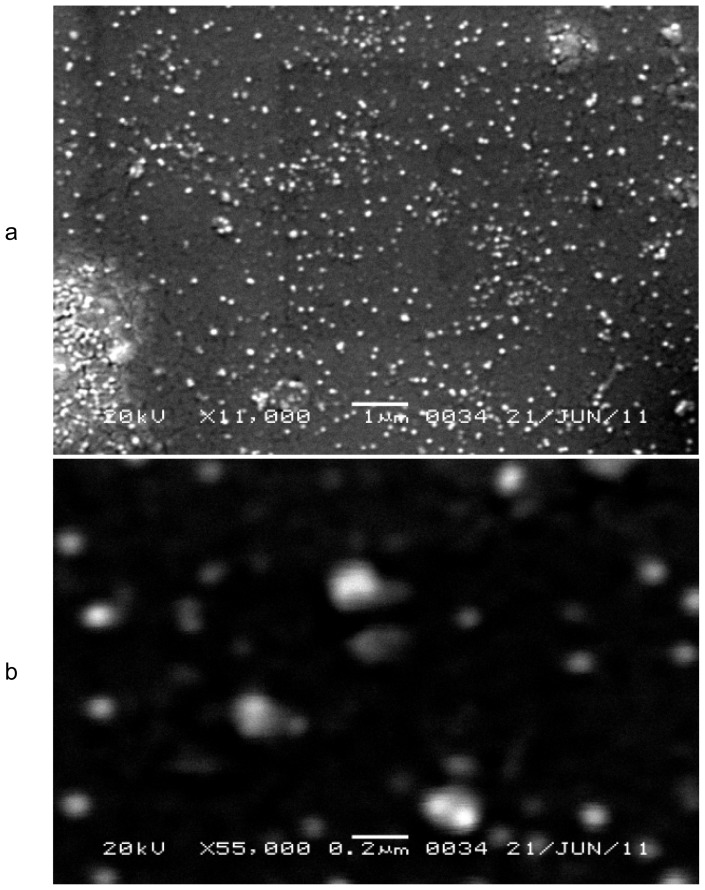
SEM images of NLC-F4 at different magnifications

Scanning electron micrographs of NLC are shown in Figure 1. The shape of the NLC was spherical and the size of the NLC was found within the nanometer range. Moreover, the micrograph also revealed the agglomeration of nanoparticles which might be due to the lipid nature of the carrier and the drying process during sample preparation prior to SEM analysis [25].

### Entrapment efficiency

The entrapment efficiency of the different formulations is presented in Table IV. The entrapment efficiency was found to be within the range of 67.195% to 77.477%, and 69.927% to 82.097% for the NLC prepared by melt-emulsification and low-temperature solidification and the high-homogenization method, respectively. The entrapment efficiency of NLC prepared by the high-speed homogenization (ultrasonication) method was found to be higher, compared to the NLC prepared by the melt-emulsification and low-temperature solidification methods.

The effect of oleic acid on drug entrapment efficiency in NLC was investigated. It has been observed that the drug entrapment efficiency of NLC had increased from 69.299 (NLC-F3) to 77.477% (NLC-F4) with an increase in the percentage of oleic acid from 15 to 30%w/w. It might be due to the incorporation of liquid lipids into solid lipids which have led to massive crystal order disturbance. Greater imperfections in the crystal lattice leave enough space to accommodate drug molecules, which ultimately improved drug-loading capacity and drug entrapment efficiency. Higher entrapment in the formulation containing 30%w/w oleic acid indicates higher solubility of aceclofenac in oleic acid, compared to stearic acid.

The effect of the amount of lecithin (phospholipon 90G) on entrapment efficiency of aceclofenac in NLC was evaluated. Table 4 reveals that the higher percentage entrapment efficiency was found, when the amount of phospholipon 90G was increased from 50 mg to 150 mg. It has been observed that increasing the phospholipid content reduces the possibility of drug loss to the external phase, and provides more space to incorporate the drug, resulting in improved entrapment efficiency. This may also be due to an excess of phospholipid possibly forming multilayers around the particle [9].

### Differential scanning calorimetry

**Fig. 2:**
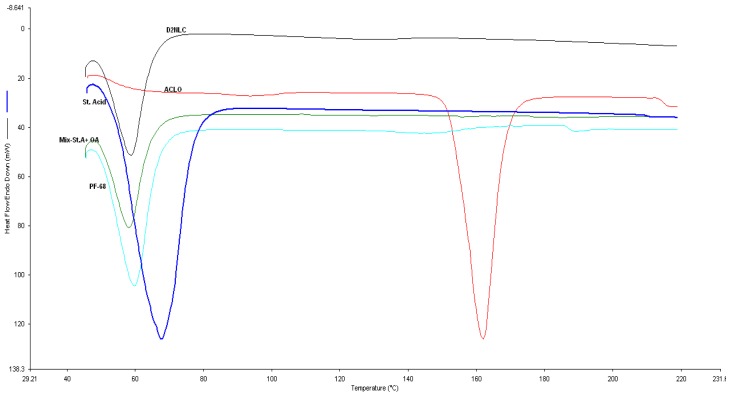
Combined DSC thermograms of Aceclofenac(ACLO), Stearic acid (SA), Physical mixture of stearic acid+ oleic acid (SA+OA), Pluronic F68 (PF68), Drug-loaded nanostructured lipid carrier (DNLC).

Figure 2 shows the results of the DSC analysis of aceclofenac, stearic acid, the physical mixture of stearic acid and oleic acid, pluronic F68, and the drug-loaded NLC formulation. From the DSC thermograms, it is observed that melting point depression occurred as the stearic acid (67.81°C) was replaced by 30% oleic acid (58.17°C). The DSC thermogram of aceclofenac showed an exothermic peak at 150.4°C, which is the reported melting point of the aceclofenac [26]. Drug-loaded NLC showed a large endothermic peak at 58.93°C. It is observed from the DSC thermogram that the exothermic peak of aceclofenac at about 150.4°C no longer exists in the DSC traces of the drug-loaded nanoparticles. Taking into consideration the drug-crystal-free particle surface, it is apparent that aceclofenac is amorphously dispersed within the nanoparticles, which is preferable to for a controlled release system. Furthermore, the inclusion of drug molecules in the lipid is normally accompanied by a depression in the lipid’s melting point [17, 27, 28].

### In vitro drug release studies of NLC dispersion

**Fig. 3:**
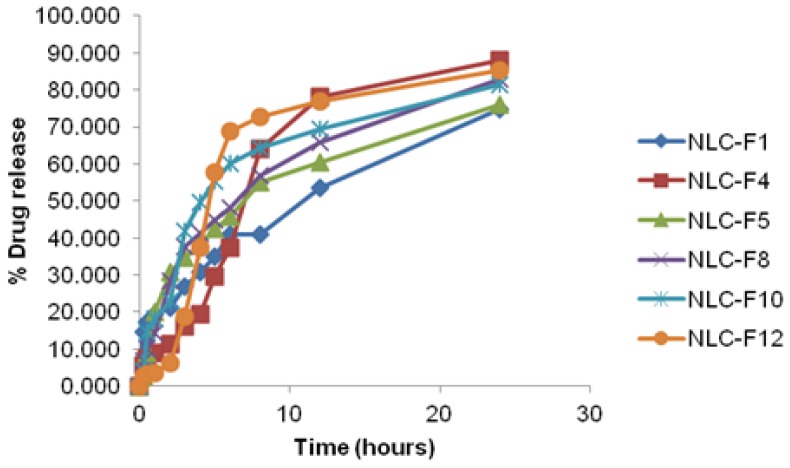
Comparative drug release profile of NLC-F1, F4, F5, F8, F10, F12 dispersion.

The *in vitro* release profile of aceclofenac from different NLC dispersions is portrayed in Figure 3. The *in vitro* release of the aceclofenac from the NLC dispersion was found to be biphasic, with the initial burst effect followed by gradual release of the aceclofenac. The initial burst release might be due to the presence of unentrapped drug in the NLC dispersion. Another reason might be due to most of the liquid lipid being located in the outer shell of the nanoparticles, which lead to a drug-enriched shell that is related to burst release at the initial stage. The oleic acid-enriched outer layers possessed a soft and considerably higher solubility for lipophilic drugs, which ultimately increased the loading of the drug and could be easily released by diffusion or matrix erosion [19]. The release rates became faster when the oleic acid concentration was increased in the NLC dispersion. The NLC dispersion containing 15%w/w of oleic acid (NLC-F5) showed a 75% release, whereas the NLC dispersion containing 30% w/w of oleic acid (NLC-F4) released up to 87% of aceclofenac within 24 hrs. This revealed that the oleic acid played an important role in the release of aceclofenac from the NLC dispersion. The initial burst of drug released varies among the different formulations and was found within the range of 2–23%. Burst release can be useful to improve the penetration of the drug, while sustained release supplies the drug over a prolonged period of time. The release profile revealed that there is no significant effect on the method of preparation on the release of aceclofenac from the NLC dispersions.

### Preparation and evaluation of aceclofenac-loaded NLC gel

Based on the particle size, the entrapment efficiency and *in vitro* release profiles of NLC dispersion**s**, with NLC-F4 and NLC-F12 formulation**s** having optimum physicochemical properties**,** were selected for the formulation of the gel for topical delivery. Lyophilized NLC powder was incorporated into the gel. The physical appearance of drug-loaded NLC gel was found to be off-white in color, smooth in texture, and translucent. All of the formulations were found to be homogenous.

### In vitro permeation study of NLC gel

**Fig. 4:**
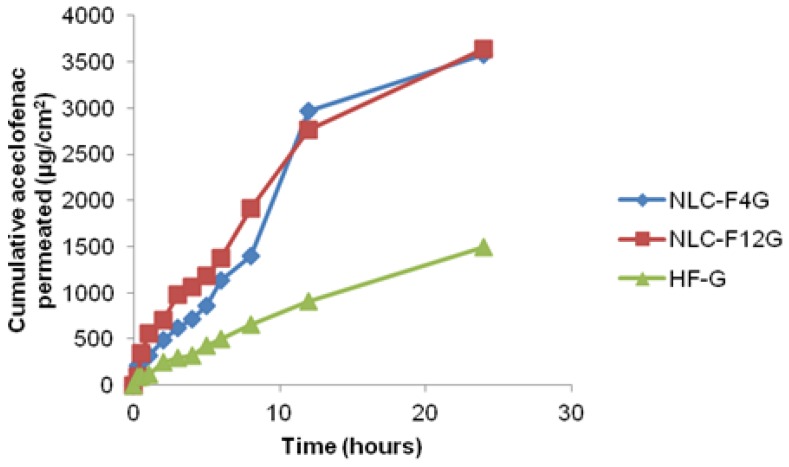
Comparative drug permeation profile of NLC-F4G, 12G, and HF-G.

*In vitro* permeation studies were performed to compare the permeation of aceclofenac to the various NLC gel formulations (NLC-F4G, NLC-F12G) and Hifenac gel (HF-G, Marketed aceclofenac gel, manufactured by Intas pharmaceuticals limited, India). The *in vitro* permeation profile is portrayed in Figure 4. All of the formulations contained the same amount of aceclofenac (1.5%w/w). The cumulative permeation profile of the different formulations revealed that the permeation of aceclofenac from the NLC gel formulation (both NLC**-**F4G and NLCF**-**12G) is greater, compared to the permeation of aceclofenac from the HF-G gel formulation. In both of the NLC**-**F4G and NLC**-**F12G formulations, 79% of aceclofenac permeated within 24 hrs, whereas 30% of aceclofenac is permeated from the HF-G formulation.

The permeation study revealed that the permeability parameters like steady-state flux (J_ss_), permeability coefficient (K_p_), and enhancement ratio were significantly higher in both of the NLC-F4G and NLC**-**F12G formulations, compared to HF-G. The cumulative amount of the permeated drug at the end of 24 hrs was 3583.194, 3641.671, and 1495.940 μg/cm^2^ with a steady-state flux (J_ss_) of 171.56, 182.22, and 68.65 μg/cm^2^/hr for NLC**-**F4G, NLC**-**F12G, and HF-G, respectively. The enhancement ratio was 2.50 and 2.65 for NLC**-**F4G and NLC**-**F12G, respectively, as compared with the HF-G formulation.

### Skin irritation study

The results of the skin irritation study revealed that following seven days’ application of NLC**-**F4G, NLC**-**F12G, HF-G, and blank gel, there was no reaction found on the skin. Therefore, it can be assured that the gel formulation can be used for topical application.

### In vivo anti-inflammatory study

The anti-inflammatory activity of the optimized formulation was evaluated by the carrageenan-induced hind paw inflammation method on Wistar rats. The percentage inhibition value of NLC-F4G and NLC-F12G was compared to HF-G. Both of the formulations NLC-F4G and NLC-F12G not only decreased the inflammation by a larger magnitude, but also sustained the effect for a prolonged period. In the first hour, the percent edema for NLC-F4G and NLC-F12G was found to be 30.59% and 32.30%, respectively (as shown in Table 5). The percentage edema inhibition for NLC-F4G and NLC-F12G was found to be 16.21% and 11.52%, respectively, while in the case of HF-G, percentage edema and percentage edema inhibition were found to be 34.09% and 6.61%, respectively. Hence the NLC-based gel formulation of aceclofenac remained superior to the marketed product in its ability to suppress edema and sustain the anti-inflammatory activity with almost double the inhibition rate after 24 hrs.

## Conclusion

This present work indicates that the NLCs of aceclofenac could be successfully prepared by the melt-emulsification and high-speed homogenization methods. The high-speed homogenization method produced smaller particles comparative with the melt-emulsification method. This study also indicates that the amount of liquid lipid and lecithin significantly affects the particle size as well as entrapment efficiency. It is also found that the release rate, permeation rate, and pharmacodynamic activity can be modulated upon changing the ratio of solid lipid to liquid lipid. It can be concluded that the optimized NLC gels exhibit faster onset and prolonged action as compared to the marketed product. Further, *in vivo* pharmacokinetic studies are necessary to assess the improvement of therapeutic efficacy of the NLC gel compared to the marketed product.

## Figures and Tables

**Tab. 1:** Composition of NLC dispersion.

Formulation code	Method of preparation	Amount of drug (%w/w)^c^	Amount of lipid		Amount of Phospholipon 90G (mg)	Amount of Pluronic F68 (%w/v)

			Stearic acid (%w/w)	Oleic acid (%w/w)		
NLC-F1/F7	Melt^a^/H.S.H^b^	10	85	15	50	1.5
NLC-F2/F8	Melt/H.S.H	10	70	30	50	1.5
NLC-F3/F9	Melt/H.S.H	10	85	15	100	1.5
NLC-F4/F10	Melt/H.S.H	10	70	30	100	1.5
NLC-F5/F11	Melt/H.S.H	10	85	15	150	1.5
NLC-F6/F12	Melt/H.S.H	10	70	30	150	1.5

a …formulations prepared by melt-emulsification and low-temperature solidification method;

b …formulations prepared by high-speed homogenization method;

c …amount of drug was calculated with respect to total lipid concentration.

**Tab. 2:** Composition of NLC-based gel formulations

Ingredients (% w/w)	Formulation		
	
	NLCF4G	NLCF12G	Blank NLCG
Carbopol 940P	1	1	1
Lyophilized NLC	1.5	1.5	–
Glycerin	10	10	10
Triethanolamine	q.s.	q.s.	q.s.
Distilled water	87.5	87.5	89.0

**Tab. 3:** Apparent partition coefficient of aceclofenac in different lipids.

System	Apparent Partition coefficient
Water/cetylpalmitate	118.45 ± 1.917
Water/stearic acid	190.84 ± 3.476
Water/tristearin	159.85 ± 1.458

Mean± SD, n= 3.

**Tab. 4:** Particle size, zeta potential and entrapment efficiency of NLC

Formulation code	Mean volume distribution (μm)			Span	Zeta Potential (mV)	%EE^a^
	d_10%_	d_50%_	d_90%_			
NLC-F1	0.126	0.173	0.284	0.913	−9.2	67.195
NLC-F2	0.129	0.186	0.280	0.812	−10.2	71.921
NLC-F3	0.129	0.183	0.276	0.803	−10.8	69.299
NLC-F4	0.126	0.171	0.234	0.632	−10.9	77.477
NLC-F5	0.126	0.173	0.260	0.775	−12.4	72.792
NLC-F6	0.127	0.175	0.246	0.680	−13.1	76.103
NLC-F7	0.126	0.171	0.286	0.936	−9.3	69.927
NLC-F8	0.126	0.171	0.274	0.865	−10.4	72.927
NLC-F9	0.126	0.173	0.253	0.734	−11.2	70.783
NLC-F10	0.126	0.170	0.237	0.653	−11.3	78.126
NLC-F11	0.132	0.199	0.268	0.683	−11.9	76.198
NLC-F12	0.128	0.173	0.233	0.607	−12.8	82.097
B-NLC(Blank)	0.125	0.173	0.257	0.763	−14.8	–

a …entrapment efficiency.

**Tab. 5:** The anti-inflammatory activity of aceclofenac loaded NLCF4G, NLCF12G, and marketed formulation (HF-G).

Group	Anti-inflammatory activity	Time (hr)						

		0	1	2	4	8	12	24
Control	Edema rate (%)	0.00 ± 0.01	36.50 ± 0.02	44.47 ± 0.02	46.79 ± 0.02	47.81 ± 0.01	43.70 ± 0.02	37.28 ± 0.02

HF-G	Edema rate (%)	0.00 ± 0.01	34.09 ± 0.02*	38.89 ± 0.02*	36.62 ± 0.03*	36.87 ± 0.02*	28.28 ± 0.02*	21.72 ± 0.02*
	Inhibition rate (%)	0	6.61	12.56	21.74	22.89	35.28	41.74

NLC**-**F4G	Edema rate (%)	0.00 ± 0.03	30.59 ± 0.03*	33.51 ± 0.02*	31.38 ± 0.03*	22.34 ± 0.02*	17.02 ± 0.01*	10.37 ± 0.03
	Inhibition rate (%)	0	16.21	24.65	32.92	53.28	61.05	72.17

NLC**-**F12G	Edema rate (%)	0.00 ± 0.02	32.30 ± 0.02*	32.04 ± 0.01*	29.97 ± 0.01*	19.12 ± 0.02*	13.44 ± 0.02*	7.49 ± 0.02*
	Inhibition rate (%)	0	11.52	27.95	35.93	60.01	69.25	79.90

* Edema rate (%) values were statistically significant from the saline control using one-way ANOVA followed by Dunnett’s t-test at p < 0.05.
